# Epidemiologic Data of Vitamin D Deficiency and Its Implication in Cardio-Cerebrovascular Risk in a Southern Italian Population

**DOI:** 10.1155/2021/5550222

**Published:** 2021-06-07

**Authors:** Rocco Capuano, Federica Marchese, Raffaella Sica, Eduardo Capuano, Marzia Manilia, Anna Grazia Iannone, Alessandro D'Ambrosio, Alvino Bisecco, Gioacchino Tedeschi, Antonio Gallo, Vincenzo Capuano

**Affiliations:** ^1^Cardiology Unit “G. Fucito” Hospital,Mercato San Severino, Azienda Ospedaliera Universitaria di Salerno, Salerno, Italy; ^2^Department of Advanced Medical and Surgical Sciences, University of Campania Luigi Vanvitelli, Napoli, Italy

## Abstract

**Background:**

Vitamin D (25(OH)D) deficiency is a prevalent condition worldwide. However, the highest prevalence rates of 25(OH)D deficiency have been attributed to regions with higher latitude. A close association between 25(OH)D and cardio-cerebrovascular (CCV) risk factors and major health problems has been identified.

**Aim:**

To establish the prevalence of 25(OH)D deficiency and to investigate the relationship between 25(OH)D levels and CCV risk factors (blood cholesterol, triglycerides, glucose concentrations, body mass index, and systolic and diastolic blood pressure) in a cohort representative of Southern Italy.

**Methods:**

The prevalence of 25(OH)D levels was evaluated in 1200 subjects aged 25–74 years (600 males and 600 females), enrolled in the “VIP” (from Italian for *Irno Valley Prevention*) Project, whereas multiple linear regression analysis was used to determine the relationship between 25(OH)D levels and CCV risk factors.

**Results:**

Only 13.3% of females and 11.1% of males showed adequate serum concentrations of 25(OH)D (≥30 ng/ml), while 59.3% of females and 55.1% of males showed 25(OH)D deficient levels (<20 ng/ml). We observed an independent association between 25(OH)D concentrations and metabolic syndrome score, LDL cholesterol, HDL cholesterol, and corrected QT (cQT).

**Conclusions:**

We report a high prevalence of 25(OH)D deficiency across the largest Italian adult population studied so far and, in particular, the first across Southern Italy; furthermore, we provide data on the association between 25(OH)D deficiency and higher CCV risk factors.

## 1. Introduction

Vitamin D plays an essential role in calcium homeostasis and bone metabolism, preventing rickets and osteomalacia [1–5]. Vitamin D is metabolized first to 25 hydroxyvitamin D (25(OH)D) and then to the hormonal form 1,25-dihydroxyvitamin D (1,25(OH)_2_D). Moved by the ubiquitous expression of 1,25(OH)_2_D receptors, the active form of vitamin D, researchers investigated the potential extraskeletal actions of vitamin D, discovering its determinant role in the autoimmune, cardiovascular, and neurological systems [6–13]. Furthermore, epidemiological studies revealed the association between low 25(OH)D serum concentrations, the surrogate marker for vitamin D nutritional status, and chronic diseases related to oxidative stress, inflammation, and aging, such as diabetes, cancer, and cardiovascular (hypertension, heart attack) and neurological (stroke, multiple sclerosis, and Alzheimer's disease) diseases [14]. As a matter of fact, research conducted on the relationship between 25(OH)D and cardiovascular events [15–27] and cerebrovascular accidents [28, 29] highlighted the association between low serum 25(OH)D concentrations and cardio-cerebrovascular (CCV) risk factors.

Adequate serum concentrations of 25(OH)D range from 30 to 100 ng/ml, while those lower than 20 ng/ml are diagnostic of vitamin D deficiency. The said condition is typically caused by inadequate cutaneous synthesis, which is, in turn, strictly related to scarce exposure to sunlight [30, 31]. To date, vitamin D deficiency is a prevalent health problem, among all ethnicities and age groups [32–35], which, hence, makes epidemiological studies essential to investigate the role of vitamin D in preventive and therapeutic strategies.

As far as Southern Italy is concerned, no epidemiological studies investigating vitamin D deficiency have been conducted among its population, although their 25(OH)D levels may be higher than those recorded in studies carried out in Northern Europe [14], due to lower latitude.

According to this background, we considered it of interest to evaluate the prevalence of vitamin D deficiency and its relationship with traditional CCV risk factors (blood cholesterol, triglycerides, glucose concentrations, body mass index, and systolic and diastolic blood pressure), in a representative Southern Italian cohort.

## 2. Methods

### 2.1. Study Design and Study Population

Data presented in this study are included in the 2018–2019 cross-sectional phase of the *Irno Valley Prevention* Project (VIP) [36], a randomized epidemiological and primary prevention study. Between January 2018 and July 2019, 1200 adults (aged 25–74 years), 600 males and 600 females, representative of the Irno Valley (Campania region), an area of Southern Italy, were randomly included from the electoral lists of two municipalities (Mercato S. Severino and Baronissi), following the rules of the Monica Project–MONICA Cardiovascular Diseases [37]. Three age and gender-matched lists of 120 subjects each were compiled. Firstly, an invitation letter was sent to the subjects in the first list. Then, in case of refusal, subjects were replaced with others from the second list and, in case of another refusal, with similar ones from the third list. The type of enrolment used allowed us to build the sample as initially planned: 120 males and 120 females per decade.

The local ethics committee approved the study, and participants provided their informed consent.

### 2.2. Data Collection and Measurement

All patients underwent a physician-administered medical history, clinical examination, electrocardiogram (ECG), and psychological assessment. Vascular risk factors and serum 25(OH)D were also measured. Body mass index (BMI) was calculated from height (in meters, using a wall altimeter) and weight (in kilograms, using a floor balance, SlimtronicRowenta®), with subjects wearing light clothes. Two consecutive measurements of blood pressure at a 5-minute interval in the sitting position were performed according to the criteria of the World Health Organization [38] and the mean used in the study. Expired carbon oxide using the Micro Smokerlyzer (Bedfont Scientific Ltd, Rochester, UK) was used to assess the exposure to cigarette smoke. Levels of anxiety and depression were evaluated using the Italian version of the State-Trait Anxiety Inventory and the Italian version of the Beck Depression Inventory—II Edition.

### 2.3. Laboratory Assays

Blood samples were obtained, after an overnight fast, from the antecubital vein without venous congestion. The enzymatic method was adopted to determine total and HDL cholesterol, triglycerides, uric acid, C-reactive protein (CRP), and glycemia. LDL cholesterol was calculated with the Friedewald equation. Serum insulin was measured by a chemiluminescent method using Beckman Coulter Diagnostics DxI 800 analyzer (interassay CV < 6%).

Creatinine was measured using the kinetics-plugged method (500 nm) on Cobas-ABX automatic line, while C3 was measured by the nephelometric method on QM 300 method. Serum 25(OH)D concentrations were measured using the enzyme-linked immunosorbent assay technique (Immunoassay System Beckman Coulter). The vitamin D nutritional status was defined according to the Endocrine Society Guidelines: (1) adequate ≥30 ng/ml, (2) insufficient ≥20 and <30 ng/ml, (3) deficient ≥10 and <20 ng/ml, and (4) severely deficient <10 ng/ml [30, 31].

### 2.4. Statistical Analysis

A Kolmogorov–Smirnov test was used to verify the normal distribution of demographic, clinical, and conventional MRI variables. The population was divided into 3 tertiles 25(OH)D:

  Group 1: 400 subjects with 25(OH)D < 15.1 ng/ml
  Group 2: 400 subjects with 25(OH)D within ≥15.1 and < 22.4 ng/ml
  Group 3: 400 subjects with 25(OH)D ≥ 22.4 ng/ml)

Across the three tertiles, we evaluated the differences in the following variables: age, height, weight, BMI, continuous metabolic syndrome score (calculated with both National Cholesterol Educational Program (NCEP) and International Diabetes Federation (IDF) methods [39]), systolic and diastolic blood pressure, HDL and LDL cholesterol, non-HDL cholesterol, triglycerides, glycemia, serum insulin, creatinine, C3, CRP, hemoglobin, white blood cells, carbon oxide, heart rate (HR), QRS, PR, QT, correct QT (cQT), depression, and anxiety. To compare quantitative variables among the 3 tertiles, we used one-way analysis of variance and the Bonferroni test. Chi-square analysis was used to test differences in prevalence. Moreover, to investigate independent associations between 25(OH)D and CCV risk factors, we carried out a multiple linear regression analysis (stepwise model), with 25(OH)D as the dependent variable adjusting for age. Lastly, we applied a sensitivity analysis, excluding subjects taking oral 25(OH)D supplements. All analyses were performed using SPSS version 20. Absolute numbers and percentages were used to describe categorical variables; means and standard deviations (SD) were used for normally distributed continuous variables.

## 3. Results

### 3.1. Prevalence of 25(OH)D Levels across Population

Baseline characteristics of study participants are reported in Table 1. Sixteen subjects (13 females and 3 males) reported taking vitamin D supplements.

Serum 25(OH)D concentrations were 19.6 ± 9.6 ng/ml in females and 20 ± 8.5 ng/ml in males (NS). No statistical difference of 25(OH)D levels was observed across different decades of age (Table 2). As reported in Table 3, only 13.3% of females and 11.1% of males had adequate concentrations of 25(OH)D (≥30 ng/ml), compared to 59.3% of females and 55.1% of males who had 25(OH)D deficient (<20 ng/ml) and severe deficient (<10 ng/ml) levels.

### 3.2. 25(OH)D Levels and CCV Risk Factors

By subdividing patients in tertiles based on the concentration of 25(OH)D (Table 4), subjects of the lowest and highest tertiles showed significant differences in BMI, metabolic syndrome, systolic blood pressure, LDL cholesterol, non-HDL cholesterol, glycemia, CRP, C3, and cQT. Furthermore, metabolic syndrome, systolic blood pressure, CRP, and glycemia were statistically different between the lowest and the intermediate tertiles, while metabolic syndrome and non-HDL cholesterol were different between the intermediate and the highest ones.

Results of the multiple linear regression analysis are shown in Table 5. When multiple regression analysis included all CCV risk factors (BMI, metabolic syndrome score, systolic blood pressure, LDL cholesterol, non-HDL cholesterol, glycemia, CRP, C3, and cQT), an independent association between 25(OH)D and NCEP metabolic syndrome score, LDL cholesterol, HDL cholesterol, and cQT was found (Table 5). In order to better understand if the burden of each variable of the metabolic syndrome score could have an independent association with 25(OH)D, we repeated the multivariate model without metabolic syndrome score and we observed a significant independent association with BMI, HDL cholesterol, non-HDL cholesterol, glycemia, and cQT (Table 6). The sensitivity analysis, carried out excluding subjects taking oral vitamin D supplements, did not modify the results obtained with the whole cohort.

## 4. Discussion

In this study, we first reported epidemiological data of serum 25(OH)D levels from the largest adult Italian population studied so far and across Southern Italy. Surprisingly, and confuting our initial hypothesis, we found a large prevalence of vitamin D deficiency among our population sample. In fact, despite the low latitude, according to studies conducted in the Northern Europe and North America [40, 41], 57.2% of our cohort showed 25(OH)D values lower than 20 ng/ml, while only 12.2% showed values between 30 and 100 ng/ml, considering the normal range by national and international guidelines [30, 31]. No significant dissimilarity of 25(OH)D levels was found between different decade groups and gender (19.6 ± 9.6 ng/ml in females and 20 ± 8.5 ng/ml in males).

These results are probably due to less sunlight exposure, independently of age and sex, mainly for social reasons and for skin cancer prevention [14], highlighting that actual sunlight exposure affects serum 25(OH)D levels more than simple latitude. Considering that vitamin D deficiency has been increasingly demonstrated to be an independent CCV risk factor, we investigated its relationship with major CCV risk factors using tertile-based categories.

Patients in the highest tertile (25(OH)D levels >22,4 ng/ml) had lower CCV risk, compared to those in the intermediate or lowest tertile, due to significantly better profiles of BMI, systolic blood pressure, metabolic syndrome, HDL cholesterol, LDL cholesterol, non-HDL cholesterol, triglycerides, glycemia, cQT, CRP, and C3. Furthermore, we found an independent association between 25(OH)D and each of the following variables: NCEP metabolic syndrome score, LDL cholesterol, HDL cholesterol, and cQT. Furthermore, removing metabolic syndrome score from the regression model, also BMI and glycemia were observed as independent predictors.

The inverse relationship between 25(OH)D and BMI has been established in various studies and, especially, in a study involving 42024 participants from 21 cohorts [14]; subsequently, it was also confirmed in cohorts of children and adolescents [42, 43] and in numerous ethnic groups [44]. A possible explanation of this finding could be the fact that obese individuals get insufficient sun exposure, perform less outdoor activities [42], and present lower 1,25(OH)_2_D receptor expression in adipose tissue [43]. Having this in mind, we can speculate that population-level interventions to reduce BMI are expected to decrease the prevalence of vitamin D deficiency; therefore, studies focused on this specific aim could be an interesting research topic. Secondly, the inverse and independent association found between 25(OH)D and glycemia is also relevant; in fact, this finding adds evidence to previous studies that showed better glycemic conditions in subjects with higher 25(OH)D levels [44, 45]. This association is particularly interesting considering that vitamin D supplementation seems to reduce type 1 diabetes mellitus incidence in childhood [46, 47] and to prevent the development and severity of peripheral neuropathy in patients with type 2 diabetes mellitus [48, 49]. These data do not support a direct cause-and-effect relationship, and indeed a recent prospective analysis provided results on the noncausal association between vitamin D and risk of type-2 diabetes, taking into account genetics as well [50]. Further studies are thus warranted.

Regarding the relationship between 25(OH)D and lipid profile, we found an inverse association between 25(OH)D levels and LDL cholesterol, non-HDL cholesterol, and a direct association between 25(OH)D and HDL [51–54], confirming previous studies. Various mechanisms have been suggested to explain the relation between 25(OH)D and lipid metabolism: (i) vitamin D and its metabolites play a role in cholesterol synthesis inhibiting 3-hydroxy-3-methyl-glutaryl-CoA-reductase and lanosterol 14a-demethylase enzyme activity in cultured cells [55]; (ii) lower LDL-C concentrations have been observed in people with higher sunlight exposure, although it is not known whether the exposure to sunlight affects cholesterol metabolism indirectly, by improving the status of vitamin D, or directly; (iii) vitamin D is essential to maintain the necessary levels of apolipoprotein A-1, the main component of HDL [56].

Finally, it is interesting to examine the indirect association between cQT and 25(OH)D levels found in our sample. In fact, very little is known about the relationship between cQT and 25(OH)D with contrasting results. Nalbant et al. demonstrated that there is no difference in cQT between people with normal levels of 25(OH)D and those with low levels of 25(OH)D [57]; on the other hand, prolonged cQT duration and cQT dispersion have been observed in patients with vitamin D deficiency [57–59].

However, our study is not without limitations: (i) the cohort studied was selected from two small municipalities in Southern Italy and could not be representative of the whole population in Italy and Southern Italy; (ii) we conducted the study in a time span of 18 months (January 2018-July 2019), with a possible impact of seasonal variations of vitamin D; (iii) due to the cross-sectional nature of the study, reverse causation could not be excluded.

In conclusion, we found a high prevalence of vitamin D deficiency across the largest Italian population studied so far; furthermore, we provided data of an association between vitamin D deficiency and higher CCV risk factors. These results provide evidence to better select the correct targeting population subgroups that may benefit from vitamin D supplementation. In fact, since now, randomized controlled trials (RCTs) have shown ambiguous results about the efficacy of vitamin D supplementation in reducing major CCV events [60–63]; this may partly be explained by taking into account the fact that heterogeneous samples were selected or that different 25(OH)D doses and analogs were used [64].

## Figures and Tables

**Table 1:** Baseline characteristics of study participants: 600 males and 600 females (aged 25–74 years).

Variables	All	Males	Females
	*N *=* *1200	*N *=* *600	*N *=* *600
25(OH)D mean ± SD (ng/ml)	19.8 ± 9.3	20 ± 8.5	19.6 ± 9.6
Weight (kg)	75.7 ± 15.7	81.9 ± 14.3	69.4 ± 14.4
Height (meters)	1.64 ± 0.1	1.71 ± 0.1	1.57 ± 0.1
Body mass index (kg/m^2^)	28.1 ± 5.3	28.1 ± 4.6	28.1 ± 5.9
Metabolic syndrome–NCEP (%)	28.8	31.7	25.8
Metabolic syndrome–IDF (%)	40.8	46.8	34.8
HDL (mg/dl)	53.8 ± 12.7	48.8 ± 11	58.7 ± 12.3
LDL (mg/dl)	123.9 ± 34.8	124.5 ± 35.7	123.2 ± 34
Non-HDL (mg/dl)	148.5 ± 38.8	152.1 ± 38.7	144.8 ± 38.7
Triglycerides (mg/dl)	123 ± 77.7	138 ± 92.4	108.1 ± 55.8
Glycemia	101.9 ± 27.6	105.2 ± 29.8	98.5 ± 24.7
Serum uric acid (mg/dl)	5.3 ± 1.5	6 ± 1.3	4.6 ± 1.2
Serum insulin (mg/dl)	9.2 ± 14.1	9 ± 10.7	9.5 ± 16.8
Creatinine (mg/dl)	1 ± 0.2	1.1 ± 0.2	0.91 ± 0.1
C3 (mg/dl)	127.4 ± 21.1	127.4 ± 19.1	127.4 ± 23
C-reactive protein (mg/L)	0.3 ± 0.6	0.3 ± 0.7	0.3 ± 0.4
Hemoglobin (g/dl)	14.1 ± 1.4	15 ± 1.1	13.3 ± 1.2
White blood cells (10^3^/*µ*L)	6.4 ± 1.6	6.6 ± 1.5	6.2 ± 1.7
Smokers	29.1%	31.2%	27%
Carbon oxide (parts per million)	3.8 ± 6.2	4.2 ± 6.6	3.3 ± 5.8
Systolic blood pressure (mmHg)	137.2 ± 20.9	143.1 ± 19	131.4 ± 20
Diastolic blood pressure (mmHg)	77.3 ± 10.9	79.6 ± 10.7	74.9 ± 10.5
Depression score	7.8 ± 7.6	6.3 ± 6.4	9.2 ± 8.4
Anxiety score	37.8 ± 10.4	35.5 ± 9.5	40 ± 10.7
Heart rate (beats/minute)	66.4 ± 10.2	65.4 ± 10.3	67.5 ± 10
QRS (milliseconds)	90.8 ± 13.1	95.2 ± 13.4	86.4 ± 11.2
PR (milliseconds)	152.4 ± 30.5	157.7 ± 35.8	147.1 ± 23.1
QT (milliseconds)	403 ± 29.8	401.1 ± 31.7	404.8 ± 27.8
cQT (milliseconds)	419.3 ± 27.3	413.1 ± 30.4	425.5 ± 22.3

Percentages were used to describe categorical variables; means and standard deviations were used for continuous variables. 25(OH)D: vitamin D; SD: standard deviation; NCEP: National Cholesterol Education Program; IDF: International Diabetes Federation; HDL: high-density lipoprotein; LDL: low-density lipoprotein.

**Table 2:** Mean and standard deviation (SD) of serum levels of 25-(OH)D (ng/ml) in females and males according to different age groups.

Age	*N*	Females	*N*	Males	*p* value
25–34	120	20.6 ± 8.1	120	19.2 ± 7.9	NS
35–44	120	20.3 ± 9.3	120	22.2 ± 7.8	NS
45–54	120	18.6 ± 9.1	120	20.1 ± 7.7	NS
55–64	120	19.2 ± 9.5	120	20 ± 10	NS
65–74	120	19.5 ± 11.5	120	18.7 ± 8.4	NS
Total	600	19.6 ± 9.6	600	20 ± 8.5	NS

*N*: number; SD: standard deviation; NS: nonsignificant.

**Table 3:** Prevalence of different vitamin D nutritional statuses: severe deficient, deficient, insufficiency, and normal values in females and males.

Reference range	Females, *N*	Prevalence (%)	Males, *N*	Prevalence (%)
Severe deficient: <10 ng/mL	80	13.3	57	9.5
Deficient: ≥10 < 20 ng/mL	276	46	273	45.6
Insufficiency: ≥20 < 30 ng/mL	164	27.4	203	33.8
Normal: ≥30 ng/mL	80	13.3	67	11.1

*N*: number.

**Table 4:** Differences of 25(OH)D tertiles in demographical, clinical, and laboratory variables.

Variables	I tertile	II tertile	III tertile	*p*	*t*		
	*N* = 400	*N* = 400	*N* = 400	—	I vs. II	I vs. III	II vs. III
25(OH)D range (ng/ml)	3.7–15.0	15.1–22.3	22.4–64.3	—	—	—	—
25(OH)D mean ± SD (ng/ml)	10.9 ± 2.6	18.5 ± 2.1	30.1 ± 7.0	—	—	—	—
Age (years)	51.5 ± 14.1	48 ± 14.1	48.4 ± 13.8	0.001	2.6^*∗*^	2.4^*∗*^	1.8
Males (%)	46.6%	49.5	52.6	NS	—	—	—

Anthropometric variables							
Weight (kg)	75.7 ± 17	76.4 ± 15.8	74.6 ± 14.3	NS	—	—	—
Height (meters)	1.63 ± 0.1	1.65 ± 0.1	1.65 ± 0.1	NS	—	—	—
Body mass index (kg/m^2^)	28.7 ± 6.0	28.2 ± 5.3	27.4 ± 4.4	0.006	1.2	3.2^*∗*^	2.0
Metabolic syndrome-NCEP (%)	34.5	31.7	20.4	<0.001	—	—	—
Metabolic syndrome-IDF (%)	44.8	42.5	35.3	0.02	—	—	—

Serum variables							
HDL cholesterol (mg/dl)	52.9 ± 12.6	53.4 ± 12.6	55.1 ± 12.8	NS	—	—	—
LDL cholesterol(mg/dl)	127 ± 35.2	125.5 ± 34.4	119.8 ± 34.8	0.02	0.6	2.7^*∗*^	2.1
Non-HDL cholesterol (mg/dl)	152.4 ± 38.7	150.5 ± 39	142.8 ± 38.2	0.003	0.6	3.2^*∗*^	2.6*∗*
Triglycerides (mg/dl)	127 ± 82.3	126.8 ± 73.9	114.8 ± 77.1	0.04	2.0	2.0	—
Glycemia	106.4 ± 36.2	100.2 ± 24.3	99.3 ± 20.2	0.001	2.9^*∗*^	3.3^*∗*^	0.4
Serum uric acid (mg/dl)	5.2 ± 1.5	5.3 ± 1.5	5.3 ± 1.4	NS	—	—	—
Serum insulin (mg/dl)	9.8 ± 15.3	10.2 ± 18	7.9 ± 6.4	NS	—	—	—
Creatinine (mg/dl)	1.0 ± 0.2	1.0 ± 0.2	1.0 ± 0.2	NS	—	—	—
C3 (mg/dl)	128.9 ± 20.9	128.7 ± 21.9	124.9 ± 20.6	0.02	0.1	2.5^*∗*^	2.3
C-reactive protein (mg/L)	0.4 ± 0.8	0.3 ± 0.4	0.3 ± 0.5	0.04	2.2^*∗*^	2.1^*∗*^	0.2
Hemoglobin (g/dl)	14.1 ± 1.5	14.1 ± 1.4	14.1 ± 1.4	NS	—	—	—
White blood cells (10^3^/*µ*/L)	6.5 ± 1.7	6.5 ± 1.6	6.3 ± 1.5	NS	—	—	

Others CCV risk factors							
Carbon oxide (parts per million)	4.4 ± 7.2	3.6 ± 5.9	3.4 ± 5.5	NS	—	—	—
Systolic blood pressure (mmHg)	140.2 ± 21.9	135.4 ± 20.9	135.7 ± 19.4	0.004	3.0^*∗*^	2.3^*∗*^	0.2
Diastolic blood pressure (mmHg)	78.3 ± 11.3	76.7 ± 10.8	76.8 ± 10.3	NS	—	—	—

Psychological variables							
Depression score	8.3 ± 7.9	7.6 ± 8	7.7 ± 6.9	NS	—	—	—
Anxiety score	38.5 ± 11.2	37.2 ± 9	37.8 ± 9.9	NS	—	—	—

ECG variables							
Heart rate (beats/minute)	67.3 ± 11.2	66.2 ± 9.4	66 ± 9.6	NS	—	—	—
QRS (milliseconds)	91 ± 15.2	90.3 ± 11.8	90.9 ± 12	NS	—	—	—
PR (milliseconds)	151.1 ± 24	152.7 ± 30.4	152.7 ± 25.1	NS	—	—	—
QT (milliseconds)	402.7 ± 30.2	403.5 ± 27.7	402.1 ± 31.3	NS	—	—	—
cQT (milliseconds)	421.6 ± 30.3	419.8 ± 22	416.5 ± 28.6	0.03	1.8	2.5^*∗*^	1.8

Absolute numbers {range (min–max)} and percentage were used to describe categorical variables; means and standard deviations (SD) were used for continuous variables. Statistical significance was accepted for values of *p* < 0.05; NS: nonsignificant; *t*: *t*-value (Bonferroni test). ^*∗*^Significant difference (*p* < 0,05) at Bonferroni test. 25(OH)D: vitamin D; NCEP: National Cholesterol Education Program; IDF: International Diabetes Federation.

**Table 5:** Multivariate analysis. Multiple linear regression model (stepwise procedure) with 25(OH)D as the dependent variable and body mass index (BMI), metabolic syndrome score of the National Cholesterol Education Program (NCEP), metabolic syndrome score of the International Diabetes Federation (IDF), systolic blood pressure, LDL cholesterol, non-HDL cholesterol, glycemia, CRP, C3, and QTc.

Variable	Beta	*p*
Metabolic syndrome score-NCEP	−0.092	0.005
QTc	−0.007	0.002
LDL cholesterol (mg/dl)	−0.094	0.001
HDL (mg/dl)	0.083	0.012

Statistical significance was accepted for values of *p* < 0.05.

**Table 6:** Multivariate analysis. Multiple linear regression model (stepwise procedure) without metabolic syndrome score: 25(OH)D as the dependent variable and body mass index (BMI), systolic blood pressure, LDL cholesterol, non-HDL cholesterol, glycemia, CRP, C3, and QTc.

Variable	Beta	*p*
BMI	−0.072	0.021
QTC	−0.066	0.026
Non-HDL cholesterol (mg/dl)	−0.094	0.001
HDL (mg/dl)	0.081	0.08
Glycemia (mg/dl)	−0.063	0.038

Statistical significance was accepted for values of *p* < 0.05.

## Data Availability

The data are stored in an internal archive, and most of the data have been shared with the NCD Risk Factor Collaboration (NCD-RisC) group.

## Abbreviations

25(OH)D: Vitamin D
CCV: Cardio-cerebrovascular
ECG: Electrocardiogram
BMI: Body mass index
NCEP: National cholesterol educational program
IDF: International Diabetes Federation
CRP: C-reactive protein
cQT: Correct QT.
